# Genomic Diversity and Selection Signatures for Weining Cattle on the Border of Yunnan-Guizhou

**DOI:** 10.3389/fgene.2022.848951

**Published:** 2022-07-07

**Authors:** Yangkai Liu, Haijian Cheng, Shikang Wang, Xiaoyv Luo, Xiaohui Ma, Luyang Sun, Ningbo Chen, Jicai Zhang, Kaixing Qu, Mingjin Wang, Jianyong Liu, Bizhi Huang, Chuzhao Lei

**Affiliations:** ^1^ Key Laboratory of Animal Genetics, Breeding and Reproduction of Shaanxi Province, College of Animal Science and Technology, Northwest A&F University, Yangling, China; ^2^ Yunnan Academy of Grassland and Animal Science, Kunming, China; ^3^ Academy of Science and Technology, Chuxiong Normal University, Chuxiong, China; ^4^ Bijie Animal Husbandry and Veterinary Science Institute, Bijie, China

**Keywords:** Weining cattle, hybrid, genetic diversity, selection signatures, UBE3D

## Abstract

Weining cattle is a Chinese indigenous breed influenced by complex breeding and geographical background. The multi-ethnic breeding culture makes Weining cattle require more attention as livestock resources for its genetic diversity. Here, we used 10 Weining cattle (five newly sequenced and five downloaded) and downloaded another 48 genome data to understand the aspects of Weining cattle: genetic diversity, population structure, and cold-adapted performance. In the current study, a high level of genetic diversity was found in Weining cattle, and its breed comprised two potential ancestries, which were *Bos taurus* and *Bos indicus*. The positive selective sweep analysis in Weining cattle was analyzed using composite likelihood ratio (CLR) and nucleotide diversity (θπ), resulting in 203 overlapped genes. In addition, we studied the cold adaptation of Weining cattle by comparing with other Chinese cattle (Wannan and Wenshan cattle) by three methods (*F*_ST_, θπ-ratio, and XP-EHH). Of the top 1% gene list, *UBE3D* and *ZNF668* were analyzed, and these genes may be associated with fat metabolism and blood pressure regulation in cold adaptation. Our findings have provided invaluable information for the development and conservation of cattle genetic resources, especially in southwest China.

## 1 Introduction

Humpless cattle (*Bos taurus*) and humped cattle (*Bos indicus*) are two main sub-species of cattle (Decker et al., 2014), and they have been directed by multiple domestication events from the early requirement of labor to the current need for beef and milk. Today, a total of 55 indigenous cattle breeds are officially identified in China. Though the hybrids of indicine × taurine breed in Chinese cattle breeds are massive in number, the composition of the ancestors remains unclear for native breeds, particularly in southwest China. In recent studies, maternal and paternal genetic markers have shown that southwest Chinese cattle were complex but interesting, which consisted of an important node for inspiring the domestication history of Chinese cattle (Lei et al., 2006; Chen et al., 2009; Xia et al., 2019). From a whole-genome aspect, cattle identified as the most purebred could be used as a nucleus for recovering the native genetic background in the current admixed population. One finding supported that domestic cattle consist of five core groups, which were European taurine, Eurasian taurine, East Asian taurine, Chinese indicine, and Indian indicine (Chen et al., 2018). Based on that information, other researchers have focused on native breeds by the whole-genome selection (WGS) studied from economic characters to adaptable ones (Kawahara-Miki et al., 2011; Kim et al., 2017; Shen et al., 2020).

To better understand the genetic basis of adapted traits in cattle, many studies have focused on kinds of breeds adapted to various environments, including Tibetan cattle at high elevations (Xin et al., 2019), Iraqi cattle in dry and hot environment (Alshawi et al., 2019), and cold acclimation to Swedish cattle breeds (Ghoreishifar et al., 2020). Herein, low-temperature stimulation can induce animal hormones and other environmental adaption, which has a direct impact on the reproduction efficiency and production level. To date, several cold environment types have been studied, and a number of candidate genes have been reported with major effects on cold adaptation in cattle. For example, *RETREG1* and *RPL7* were under strong selection in Yakut cattle, which originate from Eastern Siberia (Yurchenko et al., 2018). *FGF5*, a hair growth factor, was selected as a distinctive feature (long and dense hairs) of Yanbian cattle (Shen et al., 2020). Here, a unique alpine mountainous area with lower temperature and high humidity on the border of Yunnan–Guizhou is formed due to the uplift of the Qinghai–Tibet plateau. Few studies have been reported on the cattle adapted to cold and humid mountains in the Yunnan–Guizhou region.

Weining cattle were shaped both from multicultural zone and complex natural ecological environments. It has characteristics of rough feeding resistance, cold resistance, good climbing, and easy fattening (China National Commission of Animal Genetic Resources, 2011). Cattle in the border of Yunnan–Guizhou are a typical hybrid of *Bos taurus* × *Bos indicus*, and Weining cattle is one of them (Nie et al., 1999). Historically, multi-ethnic livestock breeding backgrounds from Yi, Miao, and Hui national minorities bred diverse native cattle breeds, which potentially contained complex genetic backgrounds in southwest China. For the long-term national autonomy management and advocating natural national culture, it makes the genetic improvement process tardy. Geographically, the uplift of altitude (2,800 m) in the Yunnan–Guizhou area caused Weining cattle to adapt to the cold (annual average temperature 10 °C) and humid (annual mean humidity 75%–80%) environment. In terms of physical characteristics, the sagging skin of the neck enhances the heat dissipation capacity of indicine cattle, but the sagging skin of Weining cattle is not developed in humid and cold environment. In the present scenario, the low socioeconomic benefits still push this cattle breed to gradually decrease (Li R. et al., 2019). Thereby, it is necessary to study the genetic diversity and adaptability of Weining cattle.

A scarce number of studies were carried out to explore the knowledge of genomic variation in Weining cattle at the genome level. We analyzed 58 whole-genome data of individuals (including five newly sequenced Weining cattle data) and identified single-nucleotide polymorphisms (SNPs) compared with those of commercial and native populations around the world based on the *Bos taurus* reference genome assembly (ARS-UCD1.2). This study may potentially reveal the ancestral components, population structure, and genetic diversity of Weining cattle.

## 2 Material and Methods

### 2.1 Ethics Statement

This study was approved by the Institutional Animal Care and Use Committee of Northwest A&F University following the recommendation of the Regulations for the Administration of Affairs Concerning Experimental Animals of China (Permit number: NWAFAC1019).

### 2.2 Sample Collection and Genome Re-sequencing

We sampled five Weining cattle from Bi’jie, Guizhou, China. These samples were collected from villages, and the farmers were interviewed in detail to ensure unrelatedness among the sampled individuals. Genomic DNA was extracted from ear tissues using the standard phenol–chloroform method (Reid, 1991). Paired-end libraries with an insert size of 500 bp were constructed for each individual, and whole-genome sequencing was performed using Illumina NovaSeq instruments at Novogene Bioinformatics Institute, Beijing, China.

In addition, another five published data of Weining cattle were downloaded, and we also downloaded genome-wide data of 48 cattle for comparison including Wenshan cattle (n = 5), Wannan cattle (n = 5), Guangfeng cattle (n = 4), Hanwoo cattle (n = 10), Brahman (n = 4), Gir (n = 2), Nelore (n = 1), Angus (n = 9), and Simmental (n = 8) (Supplementary Table S1). In total, 58 individuals were used from ten breeds in our analysis. More detailed information about all samples analyzed in this study is provided in the additional file: Supplementary Table S1. Raw FASTQ sequences have been deposited to the NCBI under the BioProject accession number PRJNA379859.

### 2.3 Read Mapping and SNP Calling

After obtaining the WGS data, all clean reads were aligned to the latest *Bos taurus* reference assembly ARS-UCD1.2 using BWA-MEM (0.7.13- r1126) with default parameters. The average mapping rate of these reads sequenced in this study was 99.28%, and the sequencing coverage was approximately 12 × per individual. Then, potential duplicate reads were filtered by Picard tools (REMOVE_DUPLICATES = true) (http://broadinstitute.github.io/picard). After that, the Genome Analysis Toolkit (GATK, version 3.8) was further used for SNP calling (McKenna et al., 2010). GATK, “variant Filtration” was implemented for all SNPs as follows: “DP < 235 (1/3-fold total sequence depth for all individuals), DP > 2,115 (3-fold of total sequence depth for all individuals), QD < 2.0, FS > 60.0, MQ < 40.0, MQRankSum < -12.5, ReadPosRankSum < -8.0 and SOR >3.0”. Finally, all the high-quality SNPs were annotated by SnpEff software (v4.3T) (Cingolani et al., 2012).

### 2.4 Population Structure and Genetic Diversity Analysis

This study used Admixture, constructed an unrooted neighbor-joining (NJ) tree, and performed PCA by using genome-wide SNPs within autosomes to determine the population genetic structure. Principal component analysis (PCA) was performed using the smartPCA program in the EIGENSOFT v5.0 software package (Patterson et al., 2006). Population structure was carried out using ADMIXTURE v1.3 with kinship (K) set from 2 to 4 (Alexander et al., 2009). NJ trees were constructed with PLINK using a pairwise genetic distance matrix and visualized with MEGA v5.0 and iTOL (v5.1.2) (https://itol.embl.de/) (Letunic and Bork, 2021).

VCFtools were used to estimate nucleotide diversity (θπ) for each breed or population (Danecek et al., 2011). The window size and step sizes were 50K and 20K bp, respectively. Linkage disequilibrium (LD) decay was calculated using PopLDdecay with default parameters (Zhang et al., 2019). Based on the number of autosomal SNPs, runs of homozygosity (ROHs) of each individual were calculated by PLINK (-homozyg-window-snp 50). We primarily calculated the total number of ROHs (0.5–1 Mb, 1–2 Mb, 2–4 Mb, and >4 Mb) per breed/population. Using ROHs to calculate the genomic inbreeding coefficient (Forutan et al., 2018), *F*_ROH_ can accurately calculate the number of inbreeding lines. *F*_ROH_ is calculated by calculating the ratio of the total length of ROH fragments in the genome to the total length ( $L_{\text{ROH}}$ ) of the genome ( $L_{\text{auto}}$ ). The formula is as follows: *F*_ROH_ = $\sum L_{\text{ROH}}/L_{\text{auto}}$ .

### 2.5 Genome-wide Selective Sweep Identification

Within Weining cattle for genome scans, we have used the nucleotide diversity (θπ) and the composite likelihood ratio (CLR), two statistic methods. θπ was estimated based on a sliding window method with windows of 50 kb and a step of 20 kb using VCFtools (Danecek et al., 2011). The CLR test was calculated for sites in non-overlapping 50-kb windows by using SweepFinder2 (Nielsen et al., 2005). The top 1% of the windows of each method was considered as candidate signatures of selection.

According to the National Cattle Resources of China (China National Commission of Animal Genetic Resources, 2011), Weining cattle is well-adapted to the cold and humid environment of Yunnan–Guizhou Plateau. We also chose Wannan cattle and Wenshan cattle as reference populations for their resistance to higher temperatures (Yan et al., 2022). To identify genomic regions of selective sweeps associated with cold adaptation, fixation index (*F*_ST_), nucleotide diversity ratio (θπ-ratio), and cross-population extended haplotype homozygosity (XP-EHH) methods were used to select positive natural regions in the Weining cattle genome. These statistics were calculated by using a sliding window approach: a 50-kb window and a step size of 20 kb. We calculated the average *F*_ST_, θπ-ratio, and XP-EHH values of each SNP window and used the outlier method to obtain the windows with the top 1% values of each method. Finally, three gene clusters of three methods in these outlier windows were carried out.

In addition, candidate genes overlapped at least in two methods were taken for Kyoto Encyclopedia of Genes and Genomes (KEGG) pathway and Gene Ontology (GO) analyses by KOBAS 3.0 (http://kobas.cbi.pku.edu.cn/) (Bu et al., 2021). Finally, the pathway terms (*p*-value <0.05) were taken as significant terms for statistics.

## 3 Results

### 3.1 Genome Sequencing, Mapping, and SNP Identification

**FIGURE 1:**
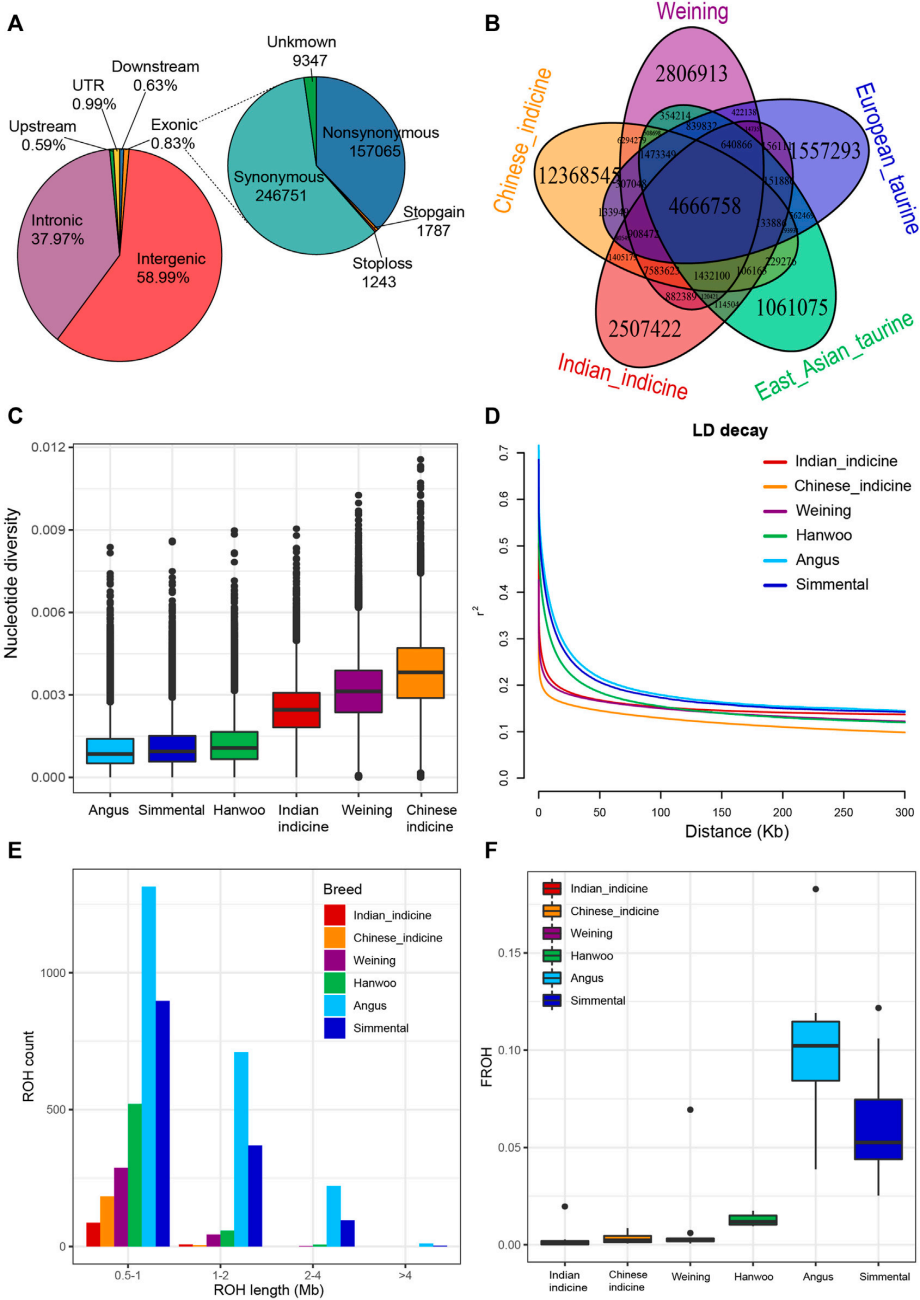
Summary statistics for genomic variation. **(A)**. Functional classification of the detected SNPs. **(B)**. Specific and shared SNPs between Weining and other cattle groups. **(C)**. Genome-wide distribution of nucleotide diversity of each breed/population in 50-kb windows with 20 kb steps. The horizontal line inside the box indicates the median of this distribution; box limits indicate the first and the third quartiles, and points show outliers. Data points outside the whiskers can be considered outliers. **(D)**. Genome-wide average LD decay estimated from each breed/population; **(E)**. Distribution of the total number of ROH across chromosomes. **(F)**. F_ROH_ of each breed/population.

Individual genomes of five Weining cattle were generated to 10.43 × coverage each and were jointly genotyped with the publicly available genome (ARS-UCD1.2) (Supplementary Table S1). To reveal the diversity of Weining cattle, 10 Weining cattle (five new and five download) were jointly genotyped with 48 publicly available genomes from four representative groups which were European taurine (Angus and Simmental), East Asian taurine (Hanwoo), Chinese indicine (Wenshan, Wannan, and Guangfeng) and Indian indicine (Nelore, Brahman, and Gir). The average alignment rate and sequencing depth of the final set reached 99.24% and ∼12 × respectively. In total, 29, 541, 306 SNPs were kept out from 10 Weining cattle genomes by snpEff. Functional annotation of the polymorphic sites revealed that the vast majority of SNPs were present in either intergenic regions (58.99%) or intronic regions (37.97%) (Supplementary Table S2). Exons contained 0.83% of the total SNPs with 157,065 non-synonymous SNPs and 246,751 synonymous SNPs (Figure 1A). Meanwhile, the analyses of specific SNPs of each breed/population have displayed that the genetic diversity of Weining cattle was second only to Chinese indicine (Figure 1B).

### 3.2 Genetic Diversity and Population Structure of Weining Cattle

The specific SNPs of each population showed a basically consistent pattern in the different populations. Specifically, Weining cattle, Chinese indicine, and Indian indicine exhibited higher specific SNP numbers, whereas the opposite genomic variations were observed in the European taurine and East Asian taurine (Figure 1B). For genomic characteristics, the nucleotide diversity of Weining (mean θπ = 0.00315) was lower than that of Chinese indicine (mean θπ = 0.0038) but approximately two times higher than that of European breeds (mean θπ = 0.001–0.0013) (Figure 1C). The genetic diversity of Weining cattle is located between indicine and taurine two clusters, but it still has higher nucleotide polymorphisms found in the indicine population (Chinese indicine > Weining cattle > Indian indicine) (Figure 1C). On the contrary, Weining cattle have a low level of LD, and the taurine population (Hanwoo, Angus, and Simmental) exhibited a higher level (Figure 1D). ROH results showed that Chinese indicine, Indian indicine, and Weining had a low self-interbreeding degree, and the main length of ROH was distributed in the interval of 0.5–1 Mb, while medium (1–2 Mb) and long (2–4 Mb) ROH fragments were found in Angus, Hanwoo, and Simmental genomes (Figure 1E). F_ROH_ also showed that Angus and Simmental cattle breeds had significantly higher inbreeding coefficients than other populations (Figure 1F).

**FIGURE 2:**
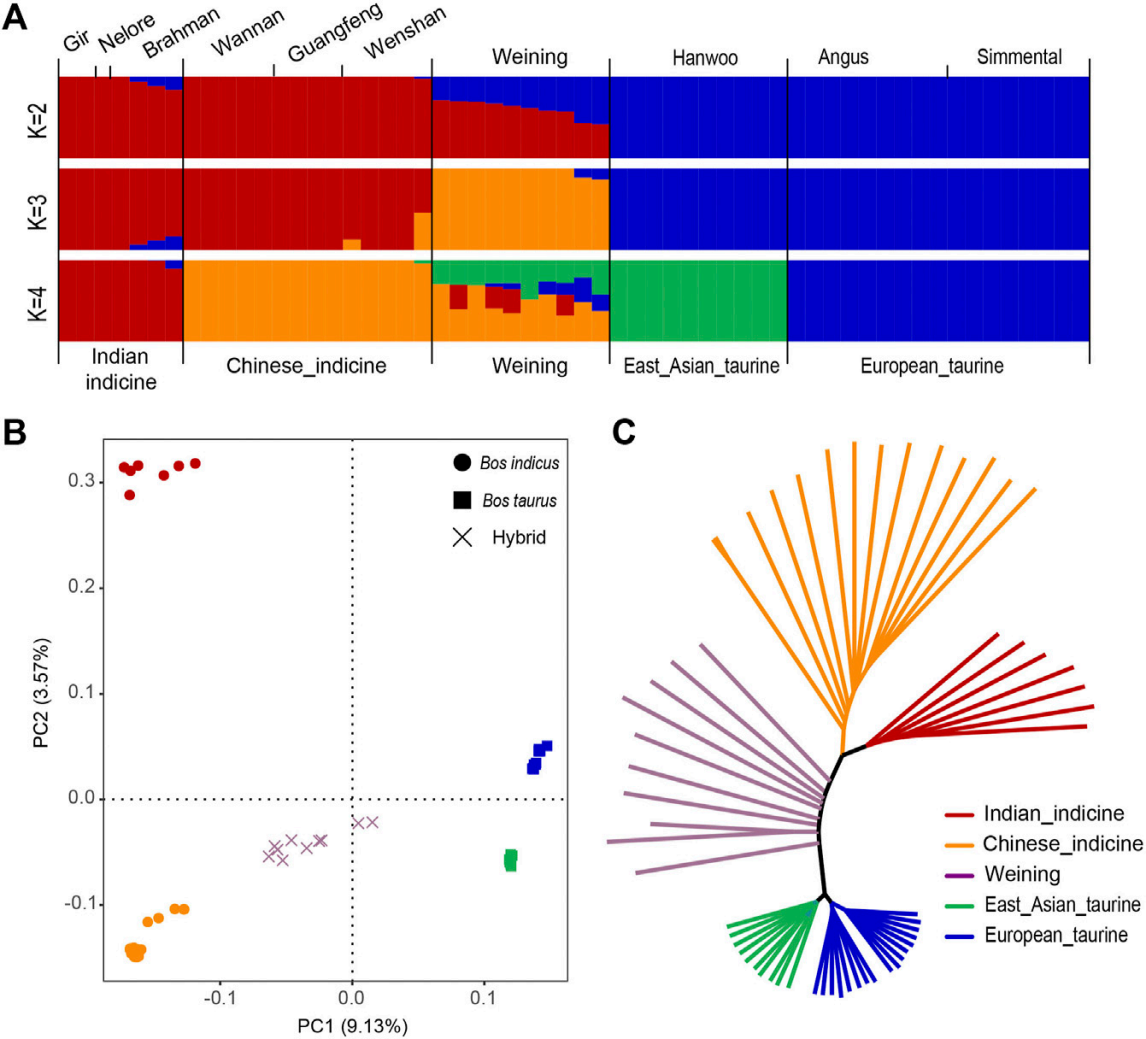
Population structure and relationships of Weining in comparison to several possible ancestral breeds. **(A)**. Model-based clustering of cattle breeds using ADMIXTURE with K = 2 and K = 4. Breeds are colored by geographic regions and labeled with breed name. **(B)**. Principal component analysis of 10 cattle breeds. **(C)**. Neighbor-joining tree of the relationships between the ten cattle breeds (58 animals).

The admixture analysis revealed two clusters (K = 2 with the lowest cross-validation error), corresponding to taurine and indicine cattle lineages (Figure 2A). Similarly, the result of PCA showed that the first PC, explaining 9.13% of the total variation, was driven by the difference between indicine and taurine cattle. The second PC, explaining 3.57% of the total variation, separated South Asian indicine cattle (Nelore, Brahman, and Gir cattle) from Chinese indicine (Wannan, Wenshan, and Guangfeng cattle) and Weining cattle (Figure 2B). The same population classification was recovered in the NJ tree (Figure 2C).

### 3.3 Genome-wide Selective Sweep Test Within Weining Breed

To further uncover genomic region information, θπ and CLR were used to detect the genome print of Weining cattle (Supplementary Figure S1A, B). Totally, 1736 and 606 genes were annotated by θπ and CLR, respectively (Supplementary Table S3, S4). After overlapping these two gene clusters, 203 genes were analyzed (Supplementary Figure S1C). Of these genes, *DLG2*, *PRLR*, *MLH1*, *CFAP299*, *GOLGA4,* and *CCNH* were reported as reproductive trait–related candidate genes (Ortega et al., 2016; Li H. et al., 2019; Singh et al., 2019; Sweett et al., 2020; Shi et al., 2021; Turan et al., 2021). The age of first mating of Weining cattle is 22 months, which is 3–5 months later than that of Wannan cattle and Wenshan cattle (China National Commission of Animal Genetic Resources, 2011). *CCNH* has been reported to be associated with male fertility in humans, and it showed low nucleotide diversity at Chr7: 87037314-87067889 of the Weining cattle genome (Supplementary Figure S1D) (Singh et al., 2019).

We also found genes (*CYB5R4*, *UBE3D,* and *VGLL2*) related to muscle growth and fat deposition (Rovadoscki et al., 2018; Hou et al., 2021; Xia et al., 2021). Furthermore, KEGG terms for overlapped genes by KOBAS were carried out, whereas, the thyroid hormone signaling pathway, PI3K-Akt signaling pathway, hippo signaling pathway, TGF-beta signaling pathway, MAPK signaling pathway, sphingolipid signaling pathway, and FoxO signaling pathway were significantly enriched (*p* < 0.05) (Supplementary Figure S1, Table S5, S6).

### 3.4 Candidate Regions and Genes Under Positive Selection in Weining Cattle Related to Cold Adaptation

**FIGURE 3:**
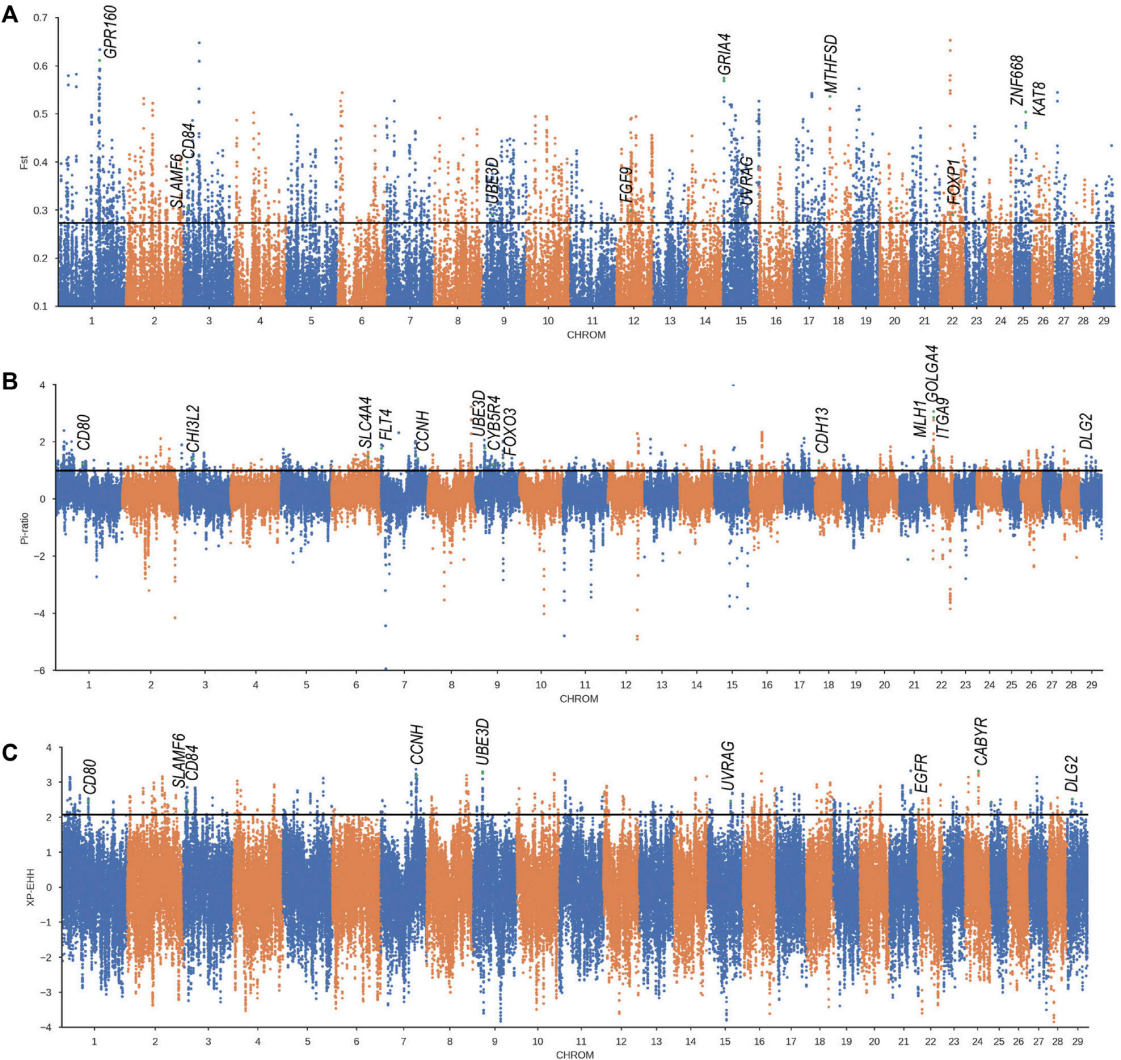
Genome-wide selection scan in Weining cattle using sliding window analysis (50 kb window size, 20 kb step size, 99th percentile cutoff) **(A)**. Selection signatures in Weining cattle for *F*_ST_ (Weining-to-Wenshan & Wannan). **(B)**. Selection signatures in Weining cattle for *π*-ratio (Wenshan & Wannan/Weining). **(C)** Selection signatures in Weining cattle for XP-EHH (Weining-to-Wenshan & Wannan). The threshold (top 1%) of *F*_ST_, *π*-ratio, and XP-EHH was marked with a horizontal black line.

**FIGURE 4:**
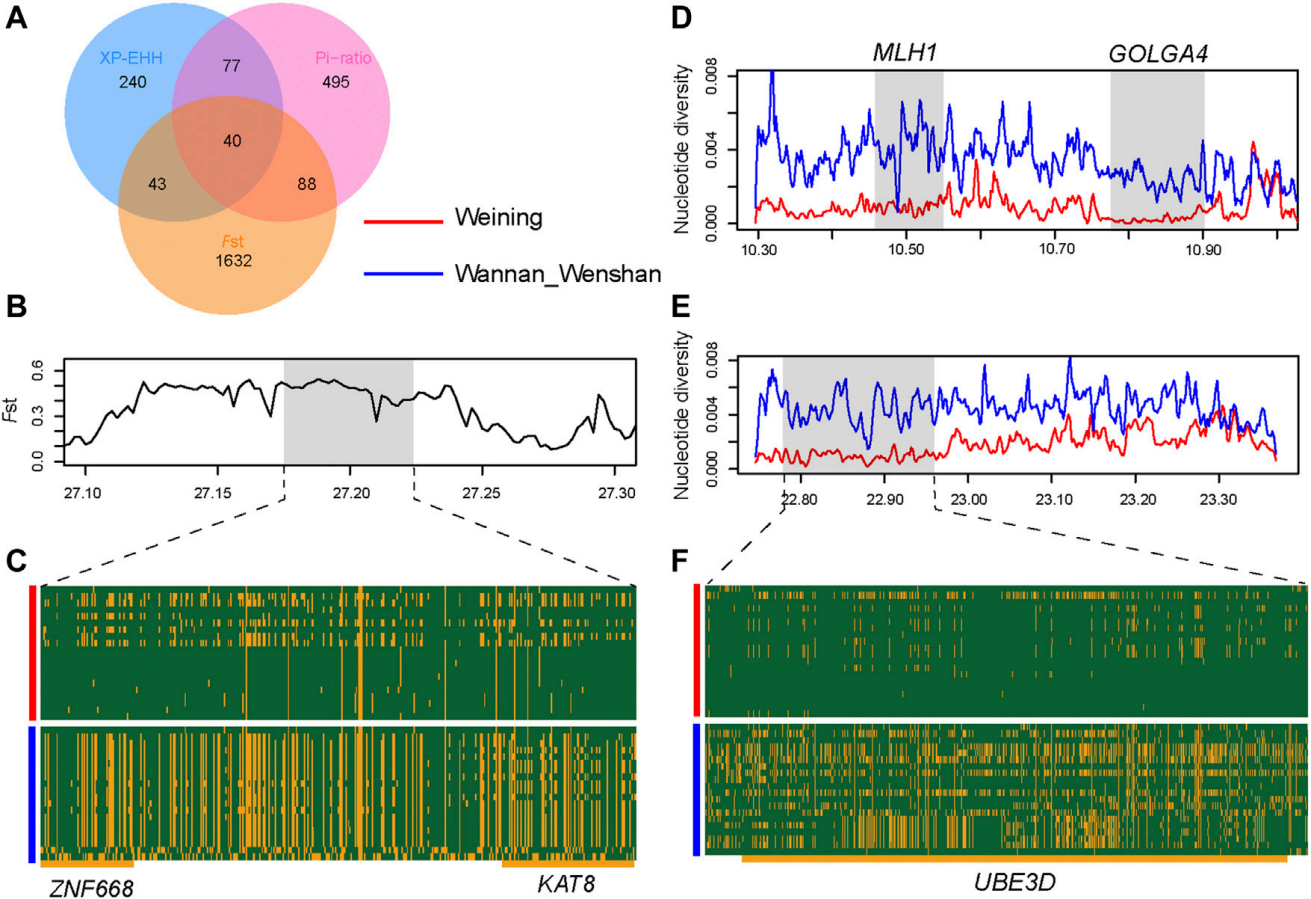
Analysis of the signatures of positive selection in the genome of Weining. **(A)** Venn diagram showing the gene overlap among θπ-ratio, *F*ST, and XP-EHH. **(B)** *F*_ST_ at the *ZNF668* and *KAT8* gene region. **(C)** SNPs with minor allele frequencies >0.05 are used to construct haplotype patterns (Chr 25: 27.17–27.22 Mb). **(D)** Nucleotide diversity plots of the *MLH1* and *GOLGA4* genomic region. **(E)** Nucleotide diversity plots of the *UBE3D* genomic region. **(F)** SNPs with minor allele frequencies > 0.05 are used to construct haplotype patterns (Chr 9: 22.77–22.96 Mb). The major allele at each SNP position in Weining is colored in yellow, and the minor one in green.

To detect the genome-wide selection signature related to cold climate adaptation, we compared Weining to the Chinese indicine population (Wannan and Wenshan cattle breeds), and significant signal regions (top 1%) were obtained by three methods (*F*_ST_, XP-EHH, and θπ-ratio) (Supplementary Table S7, S8, S9) and annotated to 1803, 400, and 700 genes respectively; of these genes, 248 genes were obtained overlapping at least in two methods (Figure 4A). To determine the most likely cold-adapted ones among these overlapped genes, we performed haplotype and non-synonymous variation analyses and reviewed lots of research studies. We found a region (Chr 9: 22800001–22850000) with high *F*_ST_, θπ-ratio, and XP-EHH values under strong selective scanning (Figure 3). This region was annotated to the *UBE3D* gene, and it also has a strong signal in CLR and θπ, which may be related to the cold adaptability of Weining cattle. In addition, *ZNF668* and *KAT8* genes were found with high *F*_ST_ values and low nucleotide diversity (Figure 4B).

In addition, KEGG and GO terms (*p* < 0.05) were obtained. KEGG pathways are enriched in terms such as “Purine metabolism, bta00230,” “MAPK signaling pathway, bta04010,” “Wnt signaling pathway, bta04310,” and “Endocrine resistance, bta01522” (Supplementary Table S10). Gene ontology (GO) terms showed that Weining cattle has increased GO categories involved in “negative regulation of chondrocyte differentiation, GO:0032331,” “positive regulation of cartilage development, GO:0061036,” “locomotory exploration behavior, GO:0035641,” and “cellular response to hormone stimulus, GO:0032870” (Supplementary Table S11).

## 4 Discussion

In this study, we analyzed 10 Weining cattle to well understand the complex population structure and high genetic diversity of Weining cattle. Mitochondrial and Y haplotypes showed that Weining cattle are a typical hybrid breed of *Bos taurus* × *Bos indicus* (Xia et al., 2019). The relatively high level of Y-chromosome variability was in accordance with the extensive mtDNA diversity in Weining cattle (Lei et al., 2006). Our result was also consistent with the non-autosomal genomic information of Weining cattle, that is, Weining cattle are hybrid of Chinese indicine and East Asian taurine, and in fact, this type of East Asian taurine and Chinese indicine dominates the crossbred type in China (Xia et al., 2021; Zhang et al., 2021). Multiple genetic backgrounds resulting from natural and artificial selection have occurred in Southwest China following the history of Chinese pastoralism. Furthermore, the pressure of rapid socioeconomic development has forced a sharp decrease in the number of groups in the Weining breed. According to breeding records, breeds such as Angus and Simmental have been introduced in the process of local breed improvement projects for the purpose of bringing more economic benefits to the locals. This partially explains the European taurine ancestry mixed into Weining cattle.

Genetic diversity in local breeds is a prerequisite for their continuous adaptation to the pressure of environmental changes. The relatively high level of genomic diversity found in Weining is likely the result of hybridization, which instills Weining cattle with not only components of *Bos taurus* but also components of *Bos indicus*. From genetic diversity analysis, Weining cattle have higher nucleotide diversity and lower inbreeding coefficient. Studies have shown that outer cattle breeds (bison, buffalo, yak, etc.) enriched the genetic diversity of Chinese indicine from the introgression (Chen et al., 2018). Interestingly, the Yunnan-Kweichow Plateau might act as an important channel for the Indian subcontinent breeds to enter China (Chen et al., 2020), making it possible for Indian indicine to mix into the genome of Weining cattle. As early as 2,500 years ago, indicus cattle extended to southern China along the east (Chen et al., 2009). More detailed information and history still need to be excavated and researched.

The fecundity of Weining cattle in mountainous areas is mainly manifested as late estrus time (China National Commission of Animal Genetic Resources, 2011), which may be caused by many factors. Research shows that the admixture of *Bos taurus* × *Bos indicus* resulted in adaptability but caused a cost of reduced reproductive fitness due to genomic incompatibility (Kim et al., 2020). Within the Weining cattle genome, we calculated CLR and θπ-ratio to analyze the positive selection regions. In chromosome 22, *MLH1*, *GOLGA4*, *DLCK3*, and *ITGA9* genes may be affected by hitchhiking effect, resulting in selection signals. Although *GOLGA4* was highly expressed in the testis of mice, a study has already shown that the knockout mice did not show relevant male fertility disorders and the *GOLGA4* gene may exist only as a redundant one (Guo et al., 2020). In the adjacent region of the *GOLGA4* gene, we found that the *MLH1* gene also had a high signal, and it was one of the candidate genes related to heifer fertility (Shi et al., 2021). A close relationship between the cold environment and the poor fertility of Weining cattle was observed. Of course, this is our conjecture, and more theoretical supports were required to experiment.

Comparing two native cattle breeds (Wannan and Wenshan cattle) bred in hot (annual average temperature 15–20 °C) and humid environments (annual mean humidity 75%–85%), we found cold-resistant candidate genes in Weining cattle, for instance: *UBE3D* and *ZNF668*. In the current study, we have selected a region including the *UBE3D* gene by all the methods (Fst, XP-EHH, and Pi-ratio). *UBE3D*, ubiquitin-protein ligase E3D, is involved in intracellular physiological processes by regulating the ubiquitination process of regulatory proteins. The study has shown that Weining cattle had high unsaturated fatty acids (UFA) (linoleic acid, alpha-Linolenic acid, etc.) in southwest Chinese native breeds (Yang and Yang, 2010). Also, the function of the ubiquitin-proteasome system (UPS) might be regulated by fatty acids physiologically (Ando et al., 2004). Coincidentally, *UBE3D* was selected as the understanding of genetic mechanisms of fat composition in sheep (Ando et al., 2004). On the other hand, the UPS maintains the stability of endoplasmic reticulum function in brown adipose tissue (BAT) by degrading useless or damaged proteins, which plays an important role in cold-adapted metabolism (Bartelt et al., 2018). In addition, a number of zinc finger genes are reported to have an association between low ambient temperature and blood pressure (Lim et al., 2017; Xu et al., 2020). *ZNF536* was related to cold tolerance in Chantecler chickens (Xu et al., 2021). Herein, methylated changes of *ZNF668* might be involved in the elevation of blood pressure to hold body temperature when exposed to a cold environment (Xu et al., 2020). Compared with other cold regions, the genetic mechanisms underlying cold tolerance in Yanbian cattle in northern China (annual average temperature 2–6°C, annual mean humidity 68.6%) might explain the parallelism at the fatty acid point in Weining cattle (Yan et al., 2022).

Weining cattle are one of the potential beef cattle breeds in southwest China. The animal breeding culture of different ethnic minorities has contributed to the hybrid of Weining cattle. Meanwhile, the rich genetic diversity causes its remarkable adaptability in cold and humid mountains. Although the impact of the market economy forces the protection and development of Weining cattle under great pressure, the diversity and enrichment of genetic information in the genome of Weining cattle is a valued material for cattle breeding.

## 5 Conclusion

Analysis of genomic diversity and selection signatures of Weining cattle were carried out at a sequence level, and new insights into the genetic basis of crossbred cattle were provided. By comparing the Weining breed with other cattle populations, we introduce its genetic diversity, population genetic structure, and environmental adaptation characteristics. Moreover, a set of candidate genes were identified and may be related to cold adaptation, low fertility, and fatty acid composition in Weining cattle, although additional physiological and functional experiments are needed for verification. Overall, it is of great significance to understand the genetic diversity and adaptation of cattle breeds in southwest China.

## Data Availability

The datasets presented in this study can be found in online repositories. The names of the repository/repositories and accession number(s) can be found in the article/Supplementary Material.

## Fundings

This work was supported by the National Natural Science Foundation of China (No. 31872317), the China Agriculture Research System of MOF and MARA (No. CARS-37), the Program of Yunling Scholar and the Young and Middle-Aged Academic Technology Leader Backup Talent Cultivation Program in Yunnan Province, China (No. 2018HB045), and the Yunnan Provincial Major S&T Project (No. 2019ZG007 and 2019ZG011).

## Supplementary Material

Supplementary Figure S1 Analysis of the signatures of selection in the genome of Weining cattle. **(A)** CLR Manhattan plot of selective sweeps in Weining cattle. **(B)** θπ Manhattan plot of selective sweeps in Weining cattle. **(C)** Venn diagram showing the genes overlap among CLR and θπ. **(D)** Nucleotide diversity plots of the *CCNH* genomic region (Chr7: 87037314-87067889). **(E)** Plot of KEGG pathway analysis of Weining cattle candidate genes overlapped by θπ and CLR methods. Threshold (top 1%) of CLR and π were marked with a horizontal black line.
