# Supplementary figures and images for: Genomic Diversity and Selection Signatures for Weining Cattle on the Border of Yunnan-Guizhou
Source: Front Genet. 2022 Jul 7;13:848951. doi: 10.3389/fgene.2022.848951 (PMC9301131; doi:10.3389/fgene.2022.848951)

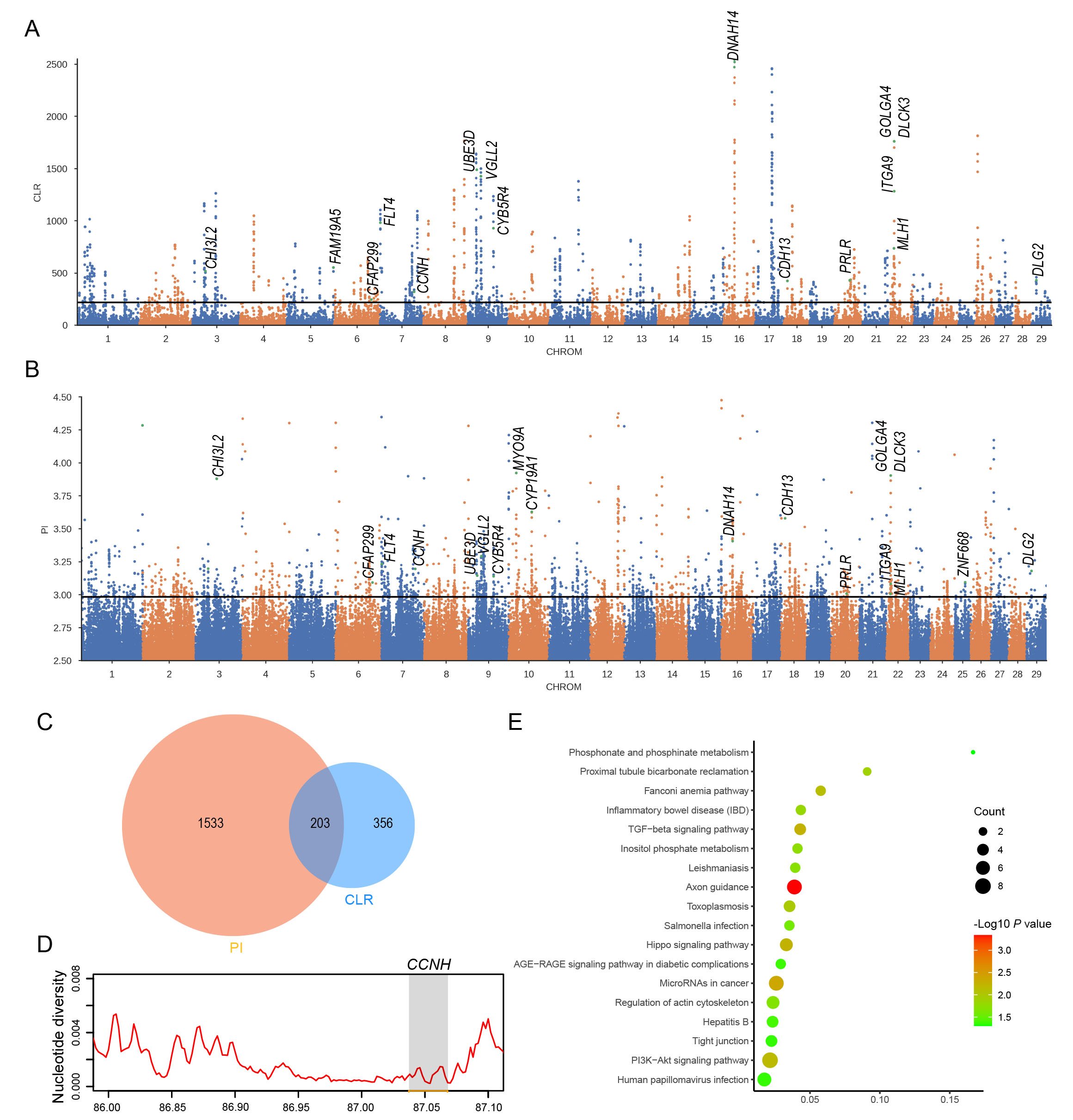

Supplement: Supplementary file 1 [file Image1.JPEG]
